# Inverse Identification of Elastic Properties of Constituents of Discontinuously Reinforced Composites

**DOI:** 10.3390/ma11112332

**Published:** 2018-11-20

**Authors:** Witold Ogierman

**Affiliations:** Institute of Computational Mechanics and Engineering, Faculty of Mechanical Engineering, Silesian University of Technology, Konarskiego 18A, 44-100 Gliwice, Poland; witold.ogierman@polsl.pl; Tel.: +48-322-372-446

**Keywords:** Mori–Tanaka model, micromechanics, homogenization, finite element analysis, random orientation

## Abstract

This paper is devoted to determination of elastic properties of composite constituents by using an inverse identification procedure. The aim of the developed identification procedure is to compute the elastic constants of individual material phases on the basis of known properties of composite materials. The inverse problem of identification has been solved by combining an evolutionary algorithm with a micromechanical model. The paper also focuses on selection of a suitable micromechanical model for optimization which should ensure a compromise between accuracy and complexity. Two different cases have been studied: composite reinforced with short cylindrical fibers and composite reinforced with cubic particles. Moreover, Monte Carlo simulations have been carried out to expose a difference in outcome of identification which may occur when uncertain input data is considered. Obtained results show that identification is successful only when properties of composite materials with at least two different volume fractions of the reinforcement are known.

## 1. Introduction

A prediction of effective properties of composite materials in terms of their microstructural features allows to design new materials in an efficient way. Micromechanical models are useful for investigation of the influence of material properties, morphology and orientation of constituents on the final, effective composite behavior. On the other hand, the mentioned quantities must be known in order to use the micromechanical models successfully. This paper focuses on determination of elastic properties of matrix and reinforcement phases. In some cases, these properties can be determined in a straightforward way by performing standard experimental tests for matrix and reinforcement materials separately. However, some composite materials are fabricated using in situ technologies; thus, quantification of elastic properties of the individual phases may be more difficult. Examples of such materials are: aluminum–aluminum oxide [1], titanium–titanium boride [2], aluminum–titanium carbide [3], cooper–titanium carbide [4] and many others. Moreover, the properties of composite constituents may change during the material processing [5,6,7]. Extraction of specimens of single phase from the composite for ex situ mechanical testing is cumbersome; therefore, in order to measure the local material properties, advanced experimental techniques like for example nanoindentation [8,9] or micropillar compression [10,11] have been developed. However, this study focuses on a different, indirect approach which is connected with the solution of an inverse problem of identification. The inverse problem of identification may be solved by combining an optimization method with a micromechanical model; in this case, microstructural quantities are variables which are adjusted in such a way that the effective material behavior predicted by the micromechanical model fits to the experimental data obtained after testing the composite. This issue has been raised by several researchers who studied different composite materials and used different methods of optimization and micromechanical models. Burczyński and Kuś [12] analyzed composites reinforced with continuous fibers; they combined finite element based homogenization with an evolutionary algorithm. Kaiser and Stommel [13] identified amorphous and crystalline constituent properties of thermoplastic material by using an evolutionary algorithm and Mori–Tanaka micromechanical model. Beluch and Burczyński [14] studied composite reinforced with continuous fibers; they applied finite element based homogenization and evolutionary algorithm as well as artificial immune system. Herrera-Solaz et al. [15] identified properties of a single crystal of AZ31 Mg alloy from polycrystal tests by combining finite element based homogenization with Levenberg–Marquard optimization method. Comellas et al. [16] studied composites reinforced with continuous fibers by using an evolutionary algorithm and mixing theory. The other way of identification of mechanical properties of composite materials presented in the literature is based on fitting the numerical model parameters to the full-field measured displacement data [17,18,19].

This paper is devoted to inverse identification of elastic constants of composite materials with randomly distributed discontinuous reinforcement. Method developed during this study assumes that elastic constants of individual material phases can be reconstructed on the basis of Young modulus and Poisson ratio of composite determined during static tensile tests. For the purpose of this study, virtual tensile tests of composites have been conducted by using the finite element method. Furthermore, the article discusses different micromechanical approaches which can be applied for the solution of the forward problem. The inverse problem has been solved by using an evolutionary algorithm. Two different cases have been studied: composite reinforced with short cylindrical fibers and composite reinforced with cubic particles. Moreover, Monte Carlo simulations have been carried out to expose a difference in outcome of identification which may occur when uncertain input data is considered. 

## 2. Identification Procedure

### 2.1. Optimization Problem

**Figure 1:**
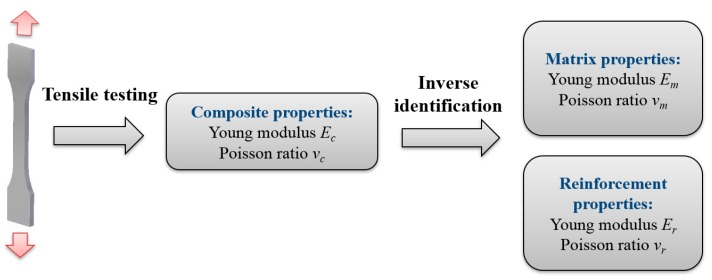
Simplified scheme presenting the idea of identification.

The identification procedure developed during this study is based on the solution of optimization problem. The objective function is defined as minimization of the relative difference between elastic constants of the composite predicted by micromechanical model in terms of constituent’s elastic constants and elastic constants of the composite determined by experimental testing:

(1) $$\min\;F(x_{1},x_{2}\ldots \;x_{k})={\sum_{i=1}^{n}\left(\frac{Y_{i}-y_{i}(x_{1},x_{2}\ldots x_{k})}{Y_{i}}\right)}^{2}$$

where: *x*_1_, *x*_2_… *x_k_* are the elastic constants of the material phases (variables), *y*_i_ are the elastic constants of the composite predicted by micromechanical model depending on variables, *Y_i_*—given elastic constants of the composite (input data), *n* denotes the number of known elastic constants of the composite. The aim of identification is presented graphically in Figure 1. 

**Figure 2:**
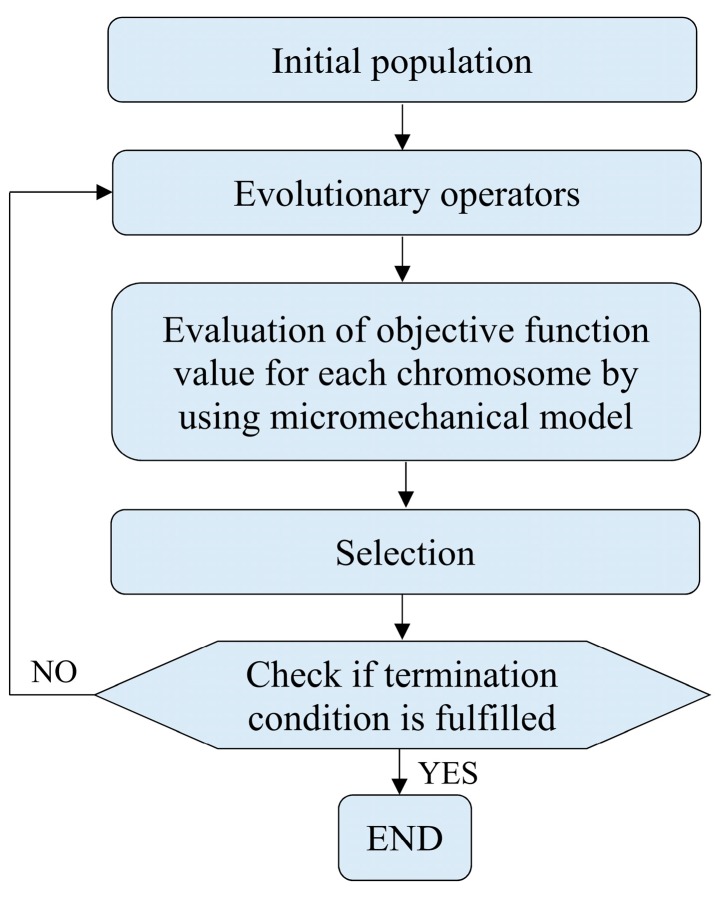
Scheme of evolutionary algorithm used for identification.

The investigation is devoted to composites with randomly distributed reinforcement; thus, isotropic effective material behavior is expected. Therefore, after the tensile testing of one coupon two elastic constants (Young modulus and Poisson ratio) can be provided as the input data. However, the input data can be extended by performing tensile tests for composites with different volume fractions of the reinforcement. During the solution of optimization problem formulated in such a way it is important to avoid getting stuck in local optimum. It could be achieved by using global optimization methods like the evolutionary algorithm [20,21,22], artificial immune algorithm [23,24] or particle swarm optimization [25,26]. Other advantages of the global optimization methods over the traditional methods are: no need of computing the objective function gradient and low impact of initial values of the project variables on the optimization results. During this work, the evolutionary algorithm implemented in MATLAB software (MathWorks, Natick, MA, USA), whose simplified scheme has been presented in Figure 2, has been applied. The first step is generation of initial population which has been chosen in random way with respect to the following optimization constraints:

(2) $$x_{i}\in \;\langle 100\;\mathrm{MPa},1000000\;\mathrm{MPa}\rangle ,$$

if the variable is Young modulus and

(3) $$x_{i}\in \;\langle 0.12,0.4\rangle .$$

if the variable is Poisson ratio. The next step is application of the evolutionary operators which are mutation and crossover, then the objective function value is computed for each individual from the population by incorporating a micromechanical model. Finally, a selection procedure is applied, and then computations are continued until the termination condition is fulfilled. In the present paper, population consisting of 50 individuals (chromosomes) has been taken into account and termination condition is fulfilled after 100 iterations (generations). 

As mentioned above, it should be pointed out that the evolutionary algorithm requires to calculate the objective function value multiple times in each iteration; therefore, efficiency of the inverse identification procedure strongly depends on efficiency of applied micromechanical model. Therefore, special emphasis must be put on the selection of a suitable micromechanical model which should ensure a compromise between accuracy and complexity. 

### 2.2. Micromechanical Modeling

Methods of micromechanics allow to predict an overall effective material properties in terms of microstructural features like for example: material properties of phases, their orientation, morphology, etc. One of the most popular methods for solving problems of micromechanics of materials is finite element analysis (FEA) which is typically connected with analysis of representative volume element (RVE) [27,28]. The RVE should contain all the necessary information about the statistical description of the microstructure, the RVE size should be large enough so that the average properties of this volume element are independent of its size and position within the material [29]. In order to find the effective material properties, homogenization procedure can be applied by computing average of field quantities over the RVE’s volume. In general, the six analyses should be performed to obtain an effective stiffness tensor by applying boundary conditions which enforce the following unit strains [30] (superscript denotes the number of analysis):

(4) $$\varepsilon^{I}=\left[\begin{array}{c} 1\\ 0\\ 0\\ 0\\ 0\\ 0 \end{array}\right],\;\varepsilon^{II}=\left[\begin{array}{c} 0\\ 1\\ 0\\ 0\\ 0\\ 0 \end{array}\right],\;\varepsilon^{III}=\left[\begin{array}{c} 0\\ 0\\ 1\\ 0\\ 0\\ 0 \end{array}\right],\;\varepsilon^{IV}=\left[\begin{array}{c} 0\\ 0\\ 0\\ 1\\ 0\\ 0 \end{array}\right],\;\varepsilon^{V}=\left[\begin{array}{c} 0\\ 0\\ 0\\ 0\\ 1\\ 0 \end{array}\right],\;\varepsilon^{VI}=\left[\begin{array}{c} 0\\ 0\\ 0\\ 0\\ 0\\ 1 \end{array}\right].$$

After FE solution of the six boundary value problems stresses are averaged in the following way:

(5) $$\langle \sigma \rangle =\frac{1}{V^{RVE}}\int_{V^{RVE}}\sigma dV^{RVE},$$

and afterwards the effective stiffness tensor can be expressed as follows:

(6) $$C=\left[\begin{array}{cccccc} {\langle \sigma_{11}\rangle }^{I} & {\langle \sigma_{11}\rangle }^{II} & {\langle \sigma_{11}\rangle }^{III} & {\langle \sigma_{11}\rangle }^{IV} & {\langle \sigma_{11}\rangle }^{V} & {\langle \sigma_{11}\rangle }^{VI}\\ {\langle \sigma_{22}\rangle }^{I} & {\langle \sigma_{22}\rangle }^{II} & {\langle \sigma_{22}\rangle }^{III} & {\langle \sigma_{22}\rangle }^{IV} & {\langle \sigma_{22}\rangle }^{V} & {\langle \sigma_{22}\rangle }^{VI}\\ {\langle \sigma_{33}\rangle }^{I} & {\langle \sigma_{33}\rangle }^{II} & {\langle \sigma_{33}\rangle }^{III} & {\langle \sigma_{33}\rangle }^{IV} & {\langle \sigma_{33}\rangle }^{V} & {\langle \sigma_{33}\rangle }^{VI}\\ {\langle \sigma_{23}\rangle }^{I} & {\langle \sigma_{23}\rangle }^{II} & {\langle \sigma_{23}\rangle }^{III} & {\langle \sigma_{23}\rangle }^{IV} & {\langle \sigma_{23}\rangle }^{V} & {\langle \sigma_{23}\rangle }^{VI}\\ {\langle \sigma_{13}\rangle }^{I} & {\langle \sigma_{13}\rangle }^{II} & {\langle \sigma_{13}\rangle }^{III} & {\langle \sigma_{13}\rangle }^{IV} & {\langle \sigma_{13}\rangle }^{V} & {\langle \sigma_{13}\rangle }^{VI}\\ {\langle \sigma_{12}\rangle }^{I} & {\langle \sigma_{12}\rangle }^{II} & {\langle \sigma_{12}\rangle }^{III} & {\langle \sigma_{12}\rangle }^{IV} & {\langle \sigma_{12}\rangle }^{V} & {\langle \sigma_{12}\rangle }^{VI} \end{array}\right]$$

The FE-based homogenization can be applied in modelling microstructures of complex geometry involving arbitrary shapes and orientation distributions of constituents; however, in such cases, it typically requires time-consuming computations [31]. Another group of micromechanical models is based on the Eshelby’s fundamental solution [32], here several approaches can be distinguished like for example self-consistent schemes [33], Mori–Tanaka method [34], double inclusion method [35]. These methods provide very efficient solution in comparison with the FE based homogenization. Particularly, the Mori–Tanaka (M-T) method found wide popularity in analysis of composite materials due to good predictive capabilities [36,37,38]. The basic formulation of M-T method provides the solution for two-phase, unidirectionally reinforced materials. In this case, the effective stiffness tensor can be determined in the following way:

(7) $$C=C_{m}+f_{i}(C_{i}-C_{m})A{\left[(1-f_{i})I+f_{i}A\right]}^{-1},$$

where C*_m_* and C*_i_* are isotropic stiffness tensors of matrix and inclusion respectively, *I* is an identity tensor, *f_i_* is volume fraction of the inclusion and *I* is strain concentration tensor that depends on the Eshelby’s tensor *S* in the following way [34]:

(8) $$A={\left[S(C_{m}{}^{-1}C_{i}-I)+I\right]}^{-1}.$$

The M-T method may be extended for composites with misaligned reinforcement by using the orientation averaging procedure where effective stiffness tensor that describes the behavior of composite with misaligned inclusions *C_ijkl_* can be determined in terms of stiffness tensor of unidirectional composite *C_pqrs_* as follows:

(9) $$C_{ijkl}=\int_{0}^{2\pi }\int_{0}^{2\pi }\int_{0}^{\pi }a_{ip}a_{jq}a_{kr}a_{ls}C_{pqrs}\psi \left(\theta ,\varphi ,\beta \right)\sin(\theta )d\theta d\varphi d\beta ,$$

where *ψ(θ,φ,β)* is the orientation distribution function defined in the Euler coordinates (*θ*,*φ*,*β*), *a_ij_* is coordinate system transformation matrix [39]. An effectiveness of the orientation-averaging procedure has been presented in numerous works [39,40,41]. The drawback of the M-T method is that it is limited to analysis of spheroidal shape of inclusions only; moreover, error of homogenization increases with increasing volume fraction of reinforcement; thus, only composites with low volume fractions of the reinforcement (approximately up to 0.25) can be successfully analyzed. However, the numerical solution of the equivalent inclusion problem, instead of using the Eshelby’s tensor, allowing the M-T method to be extended so as to involve the arbitrary shapes of the inclusions. The equivalent inclusion problem relates to analysis of single inclusion embedded in a large matrix [42]. The medium is typically approximated by a rectangular prism whose finite dimensions are large enough in comparison with the size of the inclusion [42,43]. The strain concentration tensor *A* defines the relation between the average strain in the single inclusion embedded in infinite matrix *ε^i^* and the far field strain (macro strain) *ε*:

(10) $$\left[\begin{array}{c} \varepsilon_{11}{}^{\left(i\right)}\\ \varepsilon_{22}{}^{\left(i\right)}\\ \varepsilon_{33}{}^{\left(i\right)}\\ \varepsilon_{23}{}^{\left(i\right)}\\ \varepsilon_{13}{}^{\left(i\right)}\\ \varepsilon_{12}{}^{\left(i\right)} \end{array}\right]=\left[\begin{array}{cccccc} A_{11} & A_{12} & A_{13} & A_{14} & A_{15} & A_{16}\\ A_{21} & A_{22} & A_{23} & A_{24} & A_{25} & A_{26}\\ A_{31} & A_{32} & A_{33} & A_{34} & A_{35} & A_{36}\\ A_{41} & A_{42} & A_{43} & A_{44} & A_{45} & A_{46}\\ A_{51} & A_{52} & A_{53} & A_{54} & A_{55} & A_{56}\\ A_{61} & A_{62} & A_{63} & A_{64} & A_{65} & A_{66} \end{array}\right]\left[\begin{array}{c} \varepsilon_{11}\\ \varepsilon_{22}\\ \varepsilon_{33}\\ \varepsilon_{23}\\ \varepsilon_{13}\\ \varepsilon_{12} \end{array}\right].$$

Numerical determination of components of *A* tensor is similar to the direct FE homogenization, the same boundary conditions have to be enforced (Equation (4)) although integration is performed only over the volume of the single inclusion *V^i^* as follows:

(11) $${\langle \varepsilon \rangle }^{\left(i\right)}=\frac{1}{V^{i}}\int_{V^{i}}\varepsilon^{i}dV^{i}.$$

Finally, the strain concentration tensor determined numerically has the following form:

(12) $$A^{NUM}=[\begin{array}{cccccc} {\langle \varepsilon_{11}\rangle }^{(i)I} & {\langle \varepsilon_{11}\rangle }^{(i)II} & {\langle \varepsilon_{11}\rangle }^{(i)III} & {\langle \varepsilon_{11}\rangle }^{(i)IV} & {\langle \varepsilon_{11}\rangle }^{(i)V} & {\langle \varepsilon_{11}\rangle }^{(i)VI}\\ {\langle \varepsilon_{22}\rangle }^{(i)I} & {\langle \varepsilon_{22}\rangle }^{(i)II} & {\langle \varepsilon_{22}\rangle }^{(i)III} & {\langle \varepsilon_{22}\rangle }^{(i)IV} & {\langle \varepsilon_{22}\rangle }^{(i)V} & {\langle \varepsilon_{22}\rangle }^{(i)VI}\\ {\langle \varepsilon_{33}\rangle }^{(i)I} & {\langle \varepsilon_{33}\rangle }^{(i)II} & {\langle \varepsilon_{33}\rangle }^{(i)III} & {\langle \varepsilon_{33}\rangle }^{(i)IV} & {\langle \varepsilon_{33}\rangle }^{(i)V} & {\langle \varepsilon_{33}\rangle }^{(i)VI}\\ {\langle \varepsilon_{23}\rangle }^{(i)I} & {\langle \varepsilon_{23}\rangle }^{(i)II} & {\langle \varepsilon_{23}\rangle }^{(i)III} & {\langle \varepsilon_{23}\rangle }^{(i)IV} & {\langle \varepsilon_{23}\rangle }^{(i)V} & {\langle \varepsilon_{23}\rangle }^{(i)VI}\\ {\langle \varepsilon_{13}\rangle }^{(i)I} & {\langle \varepsilon_{13}\rangle }^{(i)II} & {\langle \varepsilon_{13}\rangle }^{(i)III} & {\langle \varepsilon_{13}\rangle }^{(i)IV} & {\langle \varepsilon_{13}\rangle }^{(i)V} & {\langle \varepsilon_{13}\rangle }^{(i)VI}\\ {\langle \varepsilon_{12}\rangle }^{(i)I} & {\langle \varepsilon_{12}\rangle }^{(i)II} & {\langle \varepsilon_{12}\rangle }^{(i)III} & {\langle \varepsilon_{12}\rangle }^{(i)IV} & {\langle \varepsilon_{12}\rangle }^{(i)V} & {\langle \varepsilon_{12}\rangle }^{(i)VI} \end{array}],$$

and it could be substituted to the Equation (7) in order to find the effective stiffness tensor [44]. 

## 3. Results and Discussion

### 3.1. Composite Reinforced with Short Fibers

**Figure 3:**
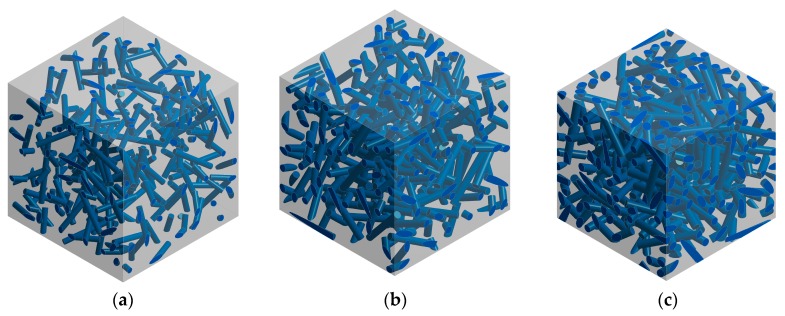
Representative volume elements representing composites reinforced with following volume fractions of cylindrical fibers: (**a**) 0.05; (**b**) 0.10; (**c**) 0.15.

Feasibility and effectiveness of the proposed inverse identification procedure has been tested by investigation of elastic constants of matrix and fiber which are part of composite material reinforced with randomly oriented short fibers. The aim of an inverse identification procedure is to reconstruct the elastic properties of matrix and fiber based on known properties of composite. Virtual tensile tests based on the FE analysis of the RVEs have been conducted for determination of composite elastic constants in terms of known elastic properties of the phases. The RVEs containing three different fiber contents, whose geometrical models are presented in Figure 3 have been considered. Each RVE contains approximately 200 fibers whose orientations are defined randomly, and periodic boundary conditions [27] have been applied. Elastic properties of matrix and fiber, which have been applied for the virtual tensile tests, are shown in Table 1. The effective elastic constants corresponding to composite properties, obtained after direct FE homogenization, are presented in Table 2.

**Figure 4:**
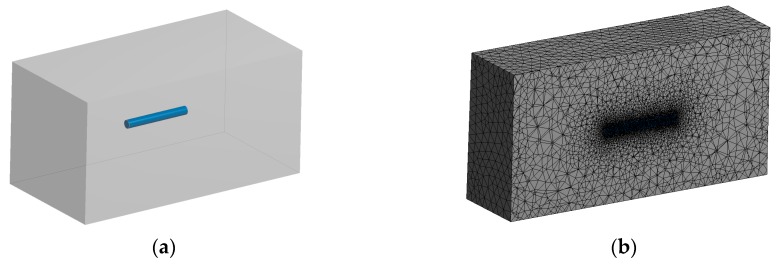
Model of equivalent inclusion problem for cylindrical fiber: (**a**) geometry; (**b**) finite element mesh.

Selection of a suitable micromechanical model for the evolutionary optimization is very important issue as pointed out in Section 2.1. The best accuracy of identification should be provided by direct FE homogenization based on the RVE containing large number of fibers (like presented in Figure 3), although it could lead to prohibitive time of computation. On the other hand, Mori–Tanaka method coupled with orientation averaging should provide relatively short time of computations, but it is not able to consider cylindrical shape of fiber and thus it must be approximated by ellipsoidal shape. Therefore, an influence of fiber shape on effective Young modulus has been tested by computing strain concentration tensor for cylindrical fiber by using FEM and comparing the result with a pure analytical solution. The geometrical model and corresponding finite element mesh of equivalent inclusion problem for cylindrical fiber have been presented in Figure 4. Single inclusion has a volume fraction 0.001 (an influence of the volume fraction on homogenization accuracy is discussed in work [45]). The following elastic constants of the composite constituents have been taken into account: *E_m_* = 10^4^ MPa, *ν_m_* = 0.3, *E_i_* = 105 MPa, *ν_i_* = 0.2.

Strain concentration tensor computed for cylindrical fiber has the following form:

(13) $$A^{NUM\_CYLINDER}=\left[\begin{array}{rrrrrr} 0.6541 & 0.0086 & 0.0086 & 0.0000 & 0.0000 & 0.0000\\ -0.0792 & 0.1644 & 0.0210 & 0.0000 & 0.0000 & 0.0000\\ -0.0792 & 0.0210 & 0.1644 & 0.0000 & 0.0000 & 0.0000\\ 0.0000 & 0.0000 & 0.0000 & 0.1756 & 0.0000 & 0.0000\\ 0.0000 & 0.0000 & 0.0000 & 0.0000 & 0.1756 & 0.0000\\ 0.0000 & 0.0000 & 0.0000 & 0.0000 & 0.0000 & 0.1430 \end{array}\right],$$

and it is in close agreement with the strain concentration tensor for ellipsoidal inclusion determined analytically in terms of Eshelby’s tensor:

(14) $$A^{ELLIPSOID}=\left[\begin{array}{rrrrrr} 0.6546 & 0.0122 & 0.0122 & 0.0000 & 0.0000 & 0.0000\\ -0.0773 & 0.1593 & 0.0207 & 0.0000 & 0.0000 & 0.0000\\ -0.0773 & 0.0207 & 0.1593 & 0.0000 & 0.0000 & 0.0000\\ 0.0000 & 0.0000 & 0.0000 & 0.1736 & 0.0000 & 0.0000\\ 0.0000 & 0.0000 & 0.0000 & 0.0000 & 0.1736 & 0.0000\\ 0.0000 & 0.0000 & 0.0000 & 0.0000 & 0.0000 & 0.1386 \end{array}\right].$$

**Figure 5:**
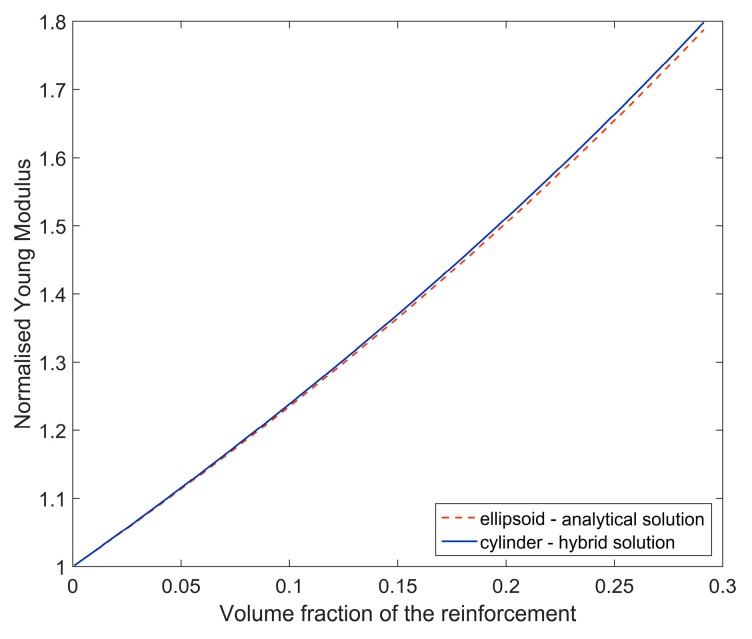
Normalized Young moduli in terms of volume fraction of the reinforcement determined by using pure analytical and hybrid method.

Finally, after the orientation averaging, normalized Young moduli obtained by basing on *A^NUM_CYLINDER^* (hybrid solution) and *A^ELLIPSOID^* (analytical solution) have been compared (Figure 5). A minor difference between the results can be noticed and, therefore, usage of the pure analytical Mori–Tanaka method (accounting for ellipsoidal shape of fiber) should provide reasonable accuracy of identification in this case. 

As an input data to the identification procedure, different combinations of known composite properties presented in Table 2 have been applied. Due to nondeterministic nature of evolutionary algorithms, three independent simulations for each case have been conducted. Results of the carried out computations and relative errors corresponding to the known data (Table 1) have been collected in Table 3.

Obtained results show that taking as input data properties of one composite material leads to inaccurate identification results, moreover each of three independent simulations gives completely different results. Taking as input data properties of two composite materials (with two different volume fractions of the reinforcement) lead to obtaining results in good agreement with prescribed, known values. In this case independent simulations give similar results. This same trend was noticed when properties of three composite materials (with three different volume fraction of the reinforcement) have been taken as an input data.

**Figure 6:**
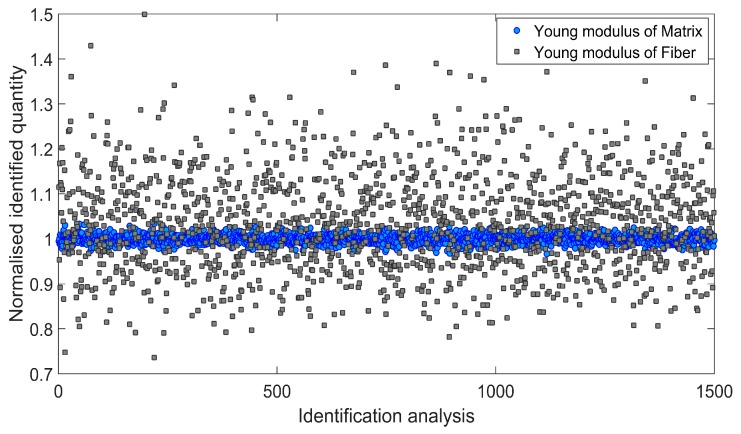
Normalized Young modulus of matrix and fiber identified during 1500 independent simulations involving standard deviation *s*_1_.

**Figure 7:**
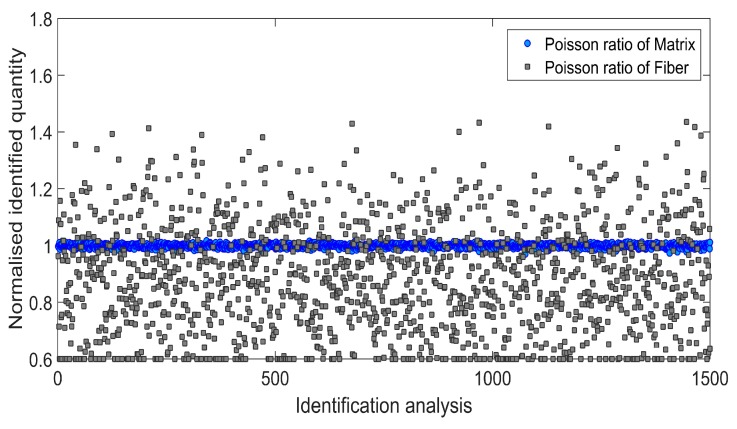
Normalized Poisson ratio of matrix and fiber identified during 1500 independent simulations involving standard deviation *s*_1_.

**Figure 8:**
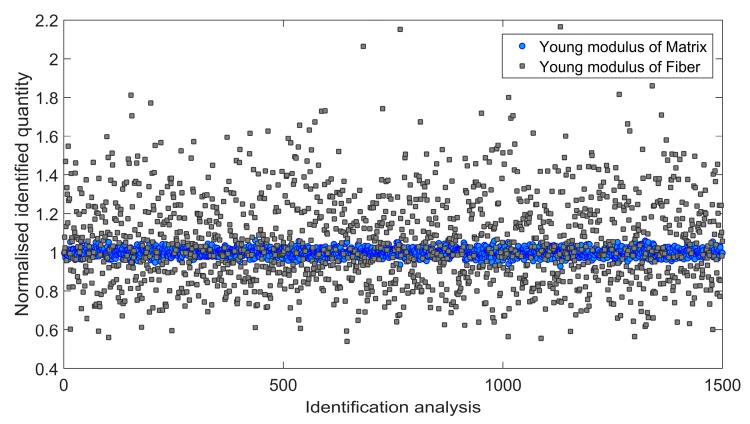
Normalized Young modulus of matrix and fiber identified during 1500 independent simulations involving standard deviation *s*_2_.

**Figure 9:**
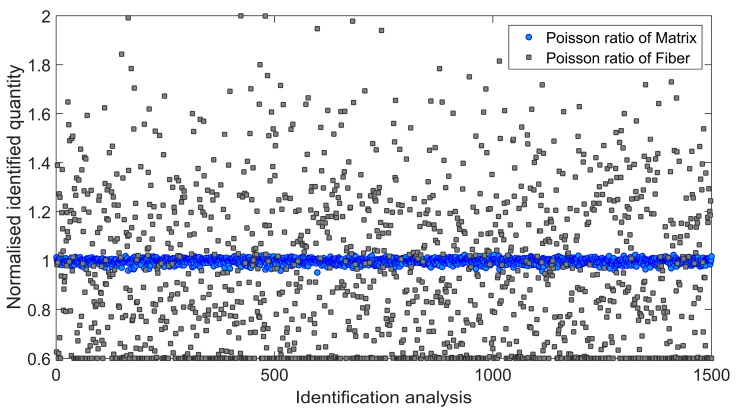
Normalized Poisson ratio of matrix and fiber identified during 1500 independent simulations involving standard deviation *s*_2_.

**Figure 10:**
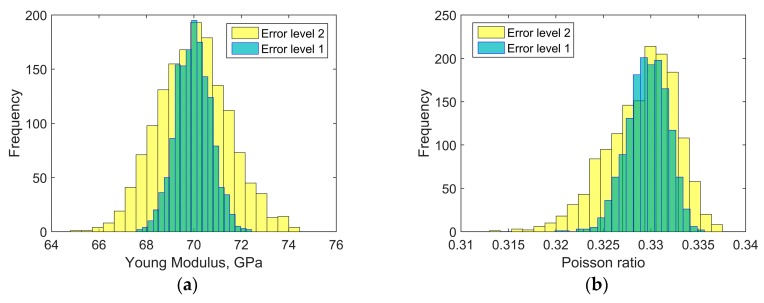

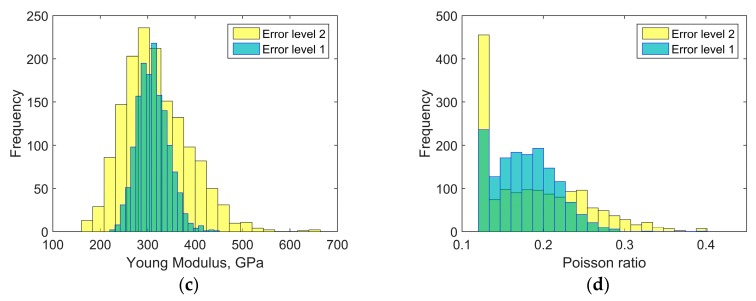
Histograms representing the distributions of identified quantities based on Monte Carlo simulations conducted for two error levels (error level 1 corresponds to the standard deviation *s*_1_, error level 2 corresponds to the standard deviation *s*_2_): (**a**) Young modulus of matrix; (**b**) Poisson ratio of matrix; (**c**) Young modulus of fiber; (**d**) Poisson ratio of fiber.

Afterwards, an influence of the input data uncertainty on identification accuracy has been investigated by performing Monte Carlo simulations [46,47]. Gaussian distribution of the input data has been taken into account by considering mean value *μ*, and two different cases of standard deviation *s*_1_ and *s*_2_ as indicated in Table 4. For each simulation, the input data was selected randomly with probability given by the gaussian distribution (1500 simulations for each standard deviation have been carried out). Monte Carlo simulations exposed a difference in outcome of identification which may occur when uncertain input data is applied. Figure 6 and Figure 7 present the results obtained for the standard deviation *s*_1_ and Figure 8 and Figure 9 present the results obtained for the standard deviation *s*_2_. Histograms that represents a distribution of identified quantities have been determined on the basis of Monte Carlo simulations (Figure 10). 

The distributions of identified elastic constants of matrix have much smaller widths than the distributions of identified elastic constants of fibers. An irregular shape of the distribution of the Poisson ratio of fibers is caused by reaching the lower optimization constraint during simulations.

### 3.2. Composite Reinforced with Cubic Particles

**Figure 11:**
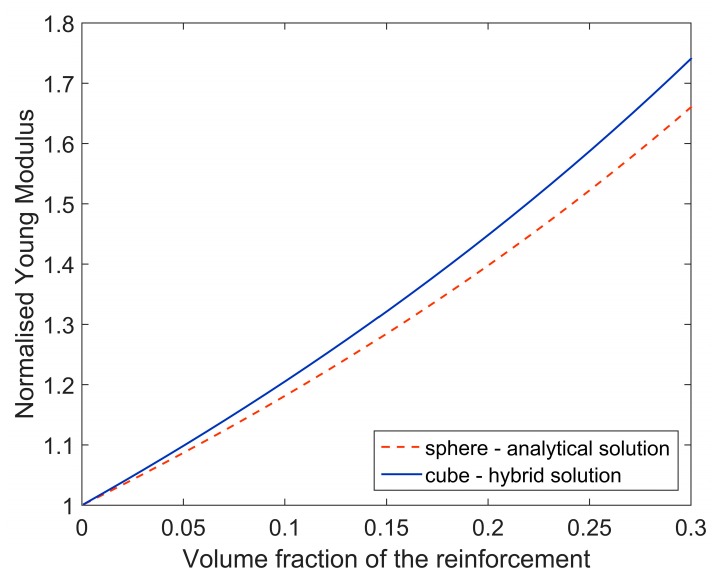
Normalized Young moduli in terms of volume fraction of the reinforcement determined by using pure analytical and hybrid method.

**Figure 12:**
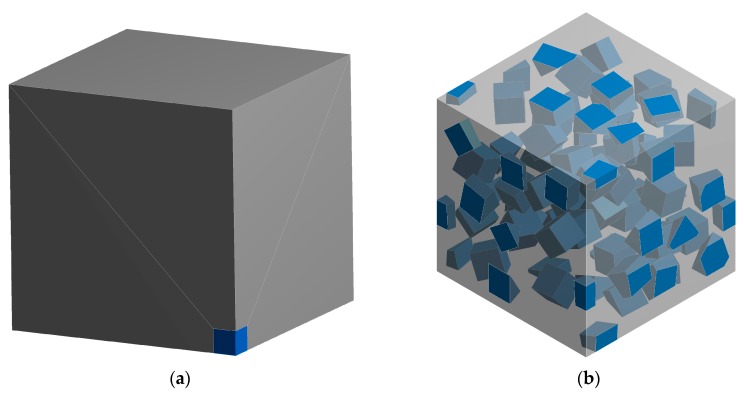
Geometrical models of: (**a**) equivalent inclusion problem for cubic particle (view on one eight of model) (**b**) representative volume element of composite reinforced with 15% of cubic particles.

Afterwards, a composite reinforced with cubic particles has been analyzed. Is this case, usage of the analytical Mori–Tanaka method may lead to inaccurate results of identification since substantial difference between Young modulus estimated by using pure analytical method (accounting for the Eshelby’s tensor for spherical inclusion) and hybrid method (involving cubic shape of inclusion) has been noticed (Figure 11). A geometrical model of equivalent inclusion problem for cubic particle is presented in Figure 12a, the following elastic constants of the composite constituents have been assumed: *E_m_* = 10^4^ MPa, *ν_m_* = 0.3, *E_i_* = 10^5^ MPa, *ν_i_* = 0.2. 

Virtual tensile tests based on the FE analysis of the RVEs have been conducted in the same way as previously (Section 3.1). The geometrical model of the RVE representing composite containing 15% of fibers is presented in Figure 12b. Elastic properties for matrix and particle are collected in Table 5. The effective elastic constants corresponding to composite material, obtained after direct FE homogenization, have been presented in Table 6.

Table 7 presents results of identification obtained by using different micromechanical models and errors corresponding to the known data. As was supposed, application of the pure analytical Mori–Tanaka method leads to substantial errors, hybrid M-F/FE approach lead to much better identification accuracy in this case. 

## 4. Concluding Remarks

This paper focused on determination of elastic properties of composite constituents by using an inverse identification procedure. The aim of the developed identification procedure is to compute the elastic constants of individual material phases on the basis of properties of composite material that may be measured experimentally. The inverse problem of identification has been solved by combining an evolutionary algorithm with a micromechanical model. Obtained results show that identification is successful only when properties of composite materials with at least two different volume fractions of the reinforcement are known, otherwise identification is ambiguous. The paper also focuses on selection of a suitable micromechanical model for optimization which should ensure a compromise between accuracy and complexity. Here, two different materials have been analyzed: in the case of composites reinforced with cylindrical fiber usage of pure analytical Mori–Tanaka method provided reasonable accuracy of identification, in the case of composite reinforced with cubic particle pure analytical method lead to substantial errors and, therefore, a hybrid homogenization method which accounts for actual reinforcement shape has to be applied. An influence of the input data uncertainty on identification accuracy has been investigated by performing Monte Carlo simulations. The Monte Carlo simulations exposed a difference in outcome of identification which may occur when input data is distributed normally. The performed numerical simulations presented the feasibility and effectiveness of proposed inverse identification procedure. On the other hand, investigation exposed identification errors that may occur, especially in the case when input data is uncertain. This study is the basis for further research connected with experimental tests.

Nonetheless, there is still a lot of work to be done to extend the identification procedure for general applications. There are several common features in engineering materials that may cause additional issues connected with the inverse identification procedure: (a) reinforcing fibers may have anisotropic properties, (b) porosity present in the material is not constant and increases with increasing volume fraction of the reinforcement, (c) orientation distribution of the reinforcement is not strictly random but depends on manufacturing process and may vary from unidirectional to random, and (d) the interface between the matrix and the reinforcement is imperfect. Moreover, the directions for future research are connected with improving the efficiency of micromechanical models for non-spheroidal inclusions, for example, by applying boundary element methods [48,49], instead of FEM, during the numerical solution of equivalent inclusion problem or developing metamodels instead of using time-consuming micromechanical models. 

## Figures and Tables

**Table 1:** Elastic properties of matrix and fiber.

Phase	Young Modulus (MPa)	Poisson Ratio
Matrix	70,000	0.33
Fiber	300,000	0.20

**Table 2:** Elastic properties of composites with three different volume fractions of fibers.

Volume Fraction of Fibers	Young Modulus (MPa)	Poisson Ratio
0.05	*E_c_^5%^* = 74,955.83	*ν_c_^5%^* = 0.32446
0.10	*E_c_^10%^* = 80,486.48	*ν_c_^10%^* = 0.31897
0.15	*E_c_^15%^* = 85,820.04	*ν_c_^15%^* = 0.31383

**Table 3:** Elastic constants of composite constituents identified by using different input data and corresponding errors.

Input Data	Analysis Number	Identified Elastic Constants			
		*E_m_*	*E_r_*	*v_m_*	*v_r_*
*E_c_^5%^, ν_c_^5%^*	1	68,383.3, 2.3%	512,943.6, 71.0%	0.33013, 0.0%	0.16642, 16.8%
	2	69,287.1, 1.0%	393,597.7, 31.2%	0.32500, 1.5%	0.38210, 91.1%
	3	72,330.0, 3.3%	152,002.5, 49.3%	0.32536, 1.4%	0.31336, 56.7%
*E_c_^10%^, ν_c_^10%^*	1	65,642.5, 6.2%	663,675.3,121.2%	0.32863, 0.4%	0.21423, 7.1%
	2	79,190.7, 13.1%	92,946.0, 69.0%	0.32298, 2.1%	0.27911, 39.6%
	3	71,523.3, 2.2%	250,707.2, 16.4%	0.32895, 0.3%	0.19039, 4.8%
*E_c_^15%^, ν_c_^15%^*	1	77,654.0, 10.9%	155,795.0, 48.1%	0.31281, 5.2%	0.32879, 64.4%
	2	62,787.5, 10.3%	709,421.2, 136.5%	0.32567, 1.3%	0.25722, 28.6%
	3	60,758.4, 13.2%	929,659.3, 209.9%	0.32381, 1.9%	0.32073, 60.4%
*E_c_^5%^, ν_c_^5%^*, *E_c_^10%^, ν_c_^10%^*	1	69,708.4, 0.4%	331,100.2, 10.4%	0.33001, 0.0%	0.16767, 16.2%
	2	69,708.4, 0.4%	331,097.8, 10.4%	0.33001, 0.0%	0.16766, 16.2%
	3	69,708.4, 0.4%	331,098.0, 10.4%	0.33001, 0.0%	0.16766, 16.2%
*E_c_^10%^, ν_c_^10%^*, *E_c_^15%^, ν_c_^15%^*	1	70,608.3, 0.9%	288,238.8, 3.9%	0.32927, 0.2%	0.18351, 8.2%
	2	70,608.3, 0.9%	288,237.8, 3.9%	0.32927, 0.2%	0.18351, 8.2%
	3	70,608.4, 0.9%	288,237.4, 3.9%	0.32927, 0.2%	0.18353, 8.2%
*E_c_^5%^, ν_c_^5%^*, *E_c_^15%^, ν_c_^15%^*	1	69,934.1, 0.1%	308,332.5, 2.8%	0.32982, 0.1%	0.17573, 12.1%
	2	69,934.1, 0.1%	308,332.6, 2.8%	0.32982, 0.1%	0.17573, 12.1%
	3	69,934.04, 0.1%	308,333.81, 2.8%	0.32982, 0.1%	0.17573, 12.1%
*E_c_^5%^, ν_c_^5%^*, *E_c_^15%^, ν_c_^15%^*, *E_c_^15%^, ν_c_^15%^*	1	70,001.2, 0.0%	308,842.0, 2.9%	0.32976, 0.1%	0.17571, 12.1%
	2	70,001.2, 0.0%	308,841.9, 2.9%	0.32976, 0.1%	0.17571, 12.1%
	3	70,001.2, 0.0%	308,841.8, 2.9%	0.32976, 0.1%	0.17571, 12.1%

**Table 4:** Statistical properties of composites reinforced with three different volume fractions of reinforcement which serves as an input data to Monte Carlo simulations.

Volume Fraction of Fibers	Young Modulus (Mpa)	Poisson Ratio
0.05	*Μ* = 74,955.83	*μ* = 0.32446
	*s*_1_ = 500.00	*s*_1_ = 0.0015
	*s*_2_ = 1000.00	*s*_2_ = 0.0030
0.10	*μ* = 80,486.48	*μ* = 0.31897
	*s*_1_ = 500.00	*s*_1_ = 0.0015
	*s*_2_ = 1000.00	*s*_2_ = 0.0030
0.15	*μ* = 85,820.04	*μ* = 0.31383
	*s*_1_ = 500.00	*s*_1_ = 0.0015
	*s*_2_ = 1000.00	*s*_2_ = 0.0030

**Table 5:** Elastic properties of matrix and particle.

Phase	Young Modulus (MPa)	Poisson Ratio
Matrix	70,000	0.30
Particle	415,000	0.16

**Table 6:** Elastic properties of composites with two different volume fractions of fibers.

Volume Fraction of Fibers	Young Modulus(MPa)	Poisson Ratio
0.10	82,012.65	0.28828
0.15	88,707.98	0.28269

**Table 7:** Identified elastic constants of particle reinforced composite constituents.

Micromechanical Model	Identified Elastic Constants			
	*E_m_*	*E_i_*	*v_m_*	*v_i_*
Mori–Tanaka (M-T)	70,180.5, 0.3%	518,546.8, 22.2%	0.29849, 0.5%	0.12000, 18.2%
Hybrid M-T/FE	70,042.9, 0.1%	432,482.4, 4.1%	0.29924, 0.3%	0.15423, 3.7%
